# Do volatile compounds produced by *Fusarium oxysporum* and *Verticillium dahliae* affect stress tolerance in plants?

**DOI:** 10.1080/21501203.2018.1448009

**Published:** 2018-03-07

**Authors:** Ningxiao Li, Seogchan Kang

**Affiliations:** a Intercollege Graduate Degree Program in Plant Biology, University Park, PA, The Pennsylvania State University, USA; b Department of Plant Pathology & Environmental Microbiology, The Pennsylvania State University, University Park, PA, USA

**Keywords:** Salt tolerance, plant defence, auxin, defence signalling, fungal volatile compounds

## Abstract

Volatile compounds (VCs) produced by diverse microbes seem to affect plant growth, development and/or stress tolerance. We investigated how VCs released by soilborne fungi *Fusarium oxysporum* and *Verticillium dahliae* affect *Arabidopsis thalian*a responses to abiotic and biotic stresses. Under salt stress, VCs from both fungi helped its growth and increased chlorophyll content. However, in contrast to wild-type *A. thaliana* (Col-0), *V. dahliae* VCs failed to increase leaf surface area in auxin signalling mutants *aux1-7, tir1-1* and *axr1-3*. Compared to wild-type Col-0, the degree of lateral root density enhanced by *V. dahliae* VCs in these mutants was also reduced. Consistent with the involvement of auxin signalling in fungal VC-mediated salt torelance, *A. thaliana* line carrying *DR5::GUS* displayed increased auxin accumulation in root apex upon exposure to *V. dahliae* VCs, and 1-naphthylphthalamic acid, an auxin transport inhibitor, adversely affected *V. dahliae* VC-mediated salt tolerance. *F. oxysporum* VCs induced the expression of *PR1* but not *PDF1.2* in *A. thaliana* lines containing *PR1::GUS* and *PFD1.2::GUS*. When challenged with *Pseudomonas syringae* after the exposure to *F. oxysporum* VCs, *A. thaliana* showed reduced disease symptoms. However, the number of bacterial cells in *F. oxysporum* VC-treated plants was not significantly different from that in control plants.

## Introduction

Even though plants have evolved multiple mechanisms to mitigate the effect of various biotic and abiotic stresses (Fujita et al. 2006; Harrison 2012; Huang et al. 2012), high levels of stress adversely affect plant growth and development, resulting in reduced crop productivity and health. Increasing salinity in certain arable lands, due to irrigation, draught or a combination of both, is one of the most serious abiotic threats to global crop production, because most crops are sensitive to high levels of NaCl (Xiong and Zhu 2002). Almost one billion hectares, representing about 7% of world’s land, are affected by high levels of salt (Metternicht and Zinck 2003). High soil salinity causes ion toxicity, osmotic and oxidative stress, and nutrient deficiency and also limits plant’s ability to uptake water from the soil (Bano and Fatima 2009). Multiple morphological, physiological and biochemical processes have been shown to be affected by salt (Munns and Tester 2008; Deinlein et al. 2014).

To ensure that the production of food, fibre and feed meets growing global demand, novel strategies for ensuring crop health in the presence of this and other types of stress are urgently needed. Deployment of evolutionarily fine-tuned beneficial plant–microbe partnerships and some of the processes underpinning their interactions has been promoted as one such strategy. Certain rhizosphere microbes are known to alleviate plant stress caused by high salinity via various mechanisms, including manipulation of plant hormone signalling (Yang et al. 2009; Shrivastava and Kumar 2015; Lenoir et al. 2016). *Achromobacter piechaudii* strain ARV8 that expresses ACC deaminase increased tomato tolerance to salt stress, presumably by reducing the production of ethylene (Mayak et al. 2004). *Trichoderma virens* and *Trichoderma atroviride* helped the growth of *Arabidopsis thaliana* under high NaCl conditions by restoring auxin homeostasis (Contreras-Cornejo et al. 2014). Volatile compounds (VCs) produced by some plant growth promoting rhizobacteria (PGPR) have been shown to alleviate plant salt stress (Zhang et al. 2008, 2010; Vaishnav et al. 2015, 2016; Ledger et al. 2016). VCs produced by *Bacillus amyloliquefaciens* GB03 conferred increased salt tolerance in *A. thaliana* presumably via tissue-specific regulation of *HKT1*, a high-affinity K^+^ transporter, and induced accumulation of osmoprotectants (Zhang et al. 2008, 2010). *Pseudomonas simiae* AU produced VCs that enhanced the growth of soybean seedlings under salt stress, and the underlying mechanism appears to include up-regulation of several salt stress-related proteins (Vaishnav et al. 2015). Similar to increased salt stress tolerance caused by VCs from PGPRs, application of individual VCs (e.g. 4-nitroguaiacol and quinoline) or a mix of VCs (e.g. 2-undecanone, 7-hexanol and 3-methylbutanol) also caused better growth under salt stress (Ledger et al. 2016; Vaishnav et al. 2016).

Besides increased salt tolerance, VCs produced by some bacteria and fungi also have been shown to elicit induced systemic resistance (ISR), a defence mechanism that protects plants from diverse pathogens (Bitas et al. 2013; Farag et al. 2013; Song and Ryu 2013; Chung et al. 2016; Li et al. 2016). For example, 1-octen-3-ol, a VC commonly produced by fungi, induced the expression of several defence-related genes in *A. thaliana*, and upon inoculation with *Botrytis cinerea* disease, symptom was less severe in 1-octen-3-ol-treated plants than control plants (Kishimoto et al. 2007). More examples of microbial VCs as potential elicitors for ISR were reviewed by Bitas et al. (2013) and Li et al. (2016).

Here, we investigated how fungal VCs affect salt tolerance and pathogen defence in plants using *A. thaliana* and soilborne fungi *Fusarium oxysporum* and *Verticillium dahliae*, the causative agents of vascular wilt diseases in diverse plants. VCs produced by both fungi have been shown to affect plant growth and development, and the underlying mechanism involves components of auxin signalling (Bitas et al. 2015; Li et al. 2018). However, how their VCs affect plant responses to (a)biotic stresses has not been studied.

## Materials and methods

### Fungal cultures and plant materials

*F. oxysporum* strains NRRL26379 and NRRL38335 were obtained from the USDA ARS Culture Collection (Peoria, IL). *V. dahliae* strains PD322 and PD413 were provided by Dr. Krishna Subbarao at University of California-Davis. These strains, preserved as conidial suspension in 20% glycerol, were revitalised by culturing on half-strength Potato Dextrose Agar (PDA; Becton, Spark, MD) at room temperature. Seeds of *A. thaliana* ecotype Col-0 were purchased from Lehle Seed Co. (Round Rock, TX). Two transgenic Col-0 lines containing *PR1::GUS* and *PDF1.2::GUS*, respectively, and auxin signalling mutants, including CS3074 (*aux1-7*), CS3075 (*axr1-3*) and CS3798 (*tir1-1*), were obtained from the Arabidopsis Biological Resource Center at the Ohio State University. Transgenic Col-0 line containing *DR5::GUS* was provided by Dr. Darrell Desveaux at University of Toronto.

### Assessment of plant salt tolerance upon fungal VC exposure

Bi-partite Petri plate (=I plate), which has been widely used for studying VC-mediated interaction between fungi and plants (Zhang et al. 2008; Paul and Park 2013; Bitas et al. 2015), was employed for this assessment. One compartment of I plate contained 12 ml half-strength PDA for culturing fungus, and the other compartment had 12 ml Murashige and Skoog (MS) medium (Sigma-Aldrich, St. Louis, MO) supplemented with no NaCl or 100 mM NaCl. Seed treatment was conducted as described in Bitas et al. (2015). *Arabidopsis* is a glycophytic plant and is sensitive to high salt throughout its development stages, especially during seed germination and seedling (Xiong and Zhu 2002), Accordingly, prior to exposing *A. thaliana* seedlings to 100 mM NaCl, they were germinated and grown on MS medium supplemented with 0.8% (w/v) agar and 0.25% (w/v) sucrose for 7 days. One plug of fungal culture (5 mm in diameter) was inoculated on the PDA side, and five Col-0 seedlings were transplanted to the MS side to initiate co-cultivation (Bitas et al. 2015). The control treatment for all experiments consisted of PDA alone (no fungus). Growth chamber conditions for co-cultivation were 22°C, 12 h light (4500 lx, 60 μmol photons/m^2^ s) and 60% relative humidity.

### Quantification of foliar and root growth

After co-cultivation for 14 days, roots were weighed immediately. Chlorophyll content was measured spectrophotometrically as previously described (Hiscox and Israelstam 1979). Leaf surface area was quantified as reported by Zhang et al. (2008). Seedlings were photographed using a digital camera (Nikon D80). Resulting images were imported into Adobe Photoshop CS4 (Adobe Systems, San Jose, CA) for measuring leaf surface area using a histogram function. Primary root length was measured by analysing photographed roots using ImageJ (http://imagej.nih.gov/ij/). The number of lateral roots was counted under stereomicroscope. Lateral root density was calculated by dividing the total number of lateral roots by primary root length.

### NPA treatment

MS medium was amended with 1-naphthylphthalamic acid (NPA, Sigma-Aldrich) to determine whether polar auxin transport is critical for mediating the effect of *V. dahliae* VCs on plant growth in the presence of 100 mM NaCl. NPA was dissolved in DMSO, and the corresponding control treatment was done on MS medium amended with the same volume of DMSO only. Both leaf surface area and lateral root density were determined.

### Assessment of GUS activity

Histochemical staining to assess GUS activity in three transgenic *A. thaliana* lines that contain *DR5::GUS, PR1::GUS* and *PDF1.2::GUS*, respectively, was performed as previously described (Bitas et al. 2015). Vacuum was applied for 5 min to seedlings in 50 mM sodium phosphate (pH 7.0), 0.1% (v/v) Triton X-100, 2 mM K_4_Fe(CN)_6_, 2 mM K_3_Fe(CN)_6_ and 2 mM X-Gluc (5-bromo-4-chloro-3-indolyl-beta-d-glucuronic acid, cyclohexylammonium salt, Gold Biotechnology, Olivette, MO). Subsequently, they were incubated for 15 h at 37°C in the dark with gentle agitation (75 rpm). Stained seedlings were cleared and dehydrated by washing with 70% ethanol solution for 30 min. After repeated washing to remove all chlorophylls, they were stored in 90% ethanol at 4°C until observation using a stereomicroscope (Olympus SZ60) and a compound microscope (Nikon 104). Images of plants were taken using Olympus DP26 camera.

### Infection of A. thaliana with P. syringae

Bacterial inoculum was prepared as previously described (Katagiri et al. 2002). *P. syringae* pathovar *tomato* (*Pst*) strain, DC3000, was cultured in King’s B (KB) medium containing kanamycin (50 mg/L) for 15 h at 200 rpm at 28°C. After washing bacterial cells collected by centrifugation twice with sterile water, they were resuspended in sterile water containing 0.02% Silwet L77 and 10 mM MgCl_2_ to the final concentration of 2 × 10^8^ colony-forming units (CFU)/mL. Seedlings of *A. thaliana* were co-cultivated with individual fungal strains for 12 days in I plate as described above. On the day of bacterial inoculation, fungal culture along with PDA was removed. Four basal leaves were inoculated with 5 μL each of the bacterial suspension. After inoculation, I plates were sealed with Parafilm and placed in the plant growth chamber for 5 days.

Disease severity was scored as the percentage of total leaf surface displaying chlorotic or necrotic symptoms, ranging from 0 (no symptom) to 100 (whole leaf affected), as previously described (Hossain et al. 2007). To determine the density of *Pst* DC3000 cells in inoculated leaves, CFU per gram of leaves was determined. Harvested leaves were weighed, surface sterilised (soaked in 70% ethanol for 15 s), rinsed thoroughly in sterile water and then homogenised in sterile water. Diluted leaf suspensions were plated on KB agar supplemented with kanamycin (50 mg/L). After 2 days of incubation at 28°C, bacterial colonies were counted.

### Statistical analysis

The experimental design was completely randomised, consisting of three replications for each treatment. Each experiment was repeated at least twice. One-way analysis of variation (ANOVA) to assess the effects of fungal VCs on growth was done using Minitab 17.3 (Minitab Inc., State College, PA). The significance of treatment was determined using the *F* value. When a significant *F* test was obtained for treatment, separation of the means was preformed using Fisher’s least significant difference test. Significance of the statistical tests was evaluated at *P* < 0.05.

## Results and discussion

### VCs emitted by both F. oxysporum and V. dahliae increased salt tolerance in A. thaliana

**Figure 1.**
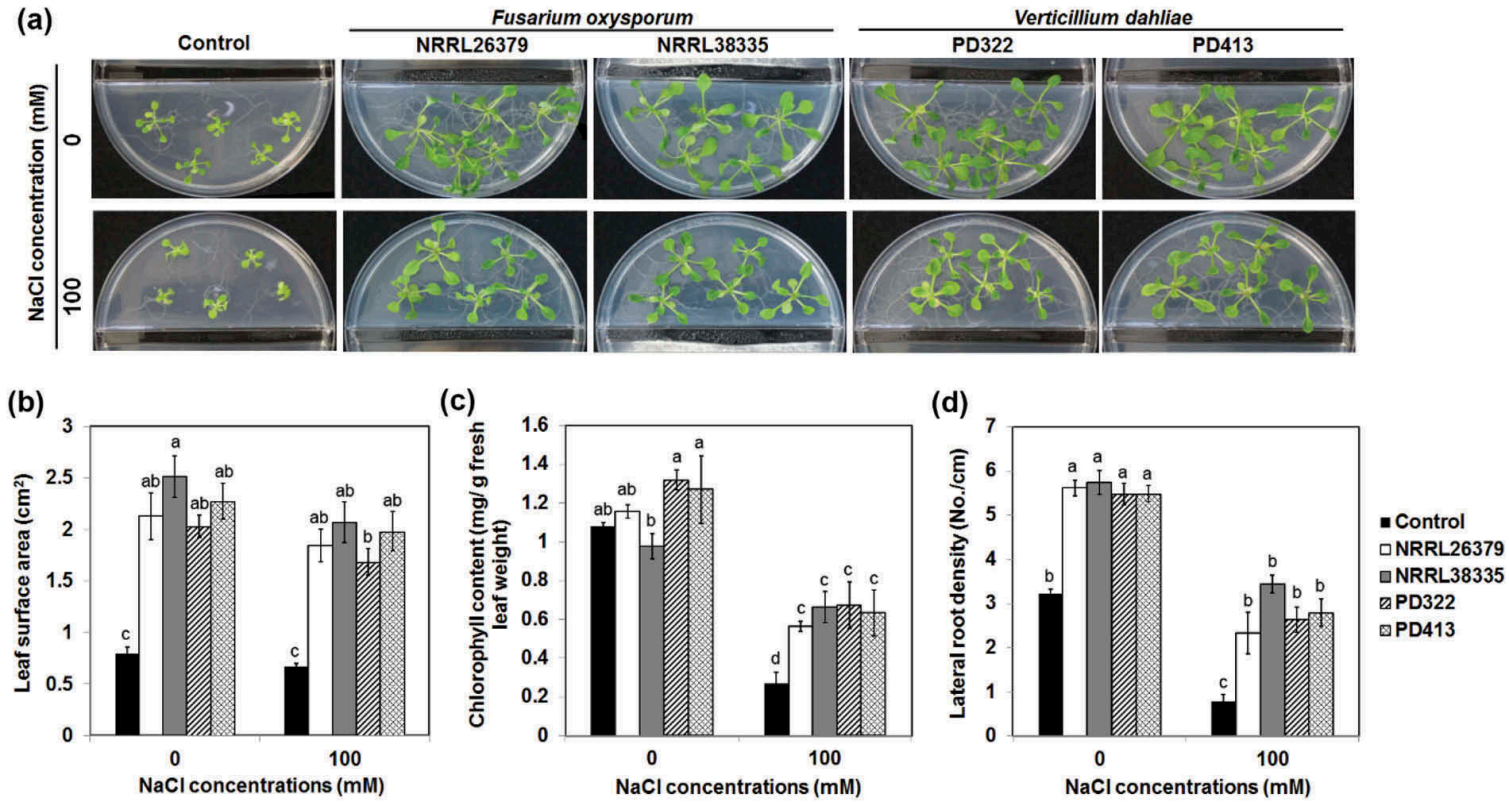
Effect of fungal VCs on *A. thaliana* in the presence of NaCl. Col-0 seedlings on medium containing no NaCl and 100 mM NaCl were co-cultivated with PDA only (control), two *F. oxysporum* strains (NRRL26379 and NRRL38335) and two *V. dahliae* strains (PD322 and PD413) for 14 days. Representative images after treatment (a), leaf surface area (b), leaf chlorophyll content (c) and lateral root density (d) are shown. Values shown correspond to the mean ± SE of data from three replicates (*n* = 15). Different letters indicate significant difference among treatments, according to Fisher’s least significant difference (LSD) test at *P* = 0.05.

To investigate how VCs produced by these fungi affect plant growth under salt stress, we employed two strains each of *F. oxysporum* (NRRL26379 and NRRL38335) and *V. dahliae* (PD322 and PD413). These strains were chosen based on the ability of their VCs to enhance plant growth and development (Bitas et al. 2015; Li et al., under revision). In agreement with our previous results, their VCs significantly enhanced the growth of *A. thaliana* in the absence of NaCl (Figure 1). In the presence of 100 mM NaCl, *A. thaliana* seedlings exposed to fungal VCs displayed much more robust foliar growth (Figure 1) than control seedlings. In addition, VC-treated plants had increased leaf chlorophyll content (Figure 1(c)) and lateral root density (Figure 1(d)). Similarly, VCs produced by certain PGPR strains also improved plant growth under salt stress (Zhang et al. 2008, 2010; Vaishnav et al. 2015, 2016; Ledger et al. 2016). Decreased leaf surface area and chlorophyll content are common plant responses to salt stress, resulting in reduced photosynthesis and growth (Netondo et al. 2004; Hasanuzzaman et al. 2013; Negrão et al. 2017). The ability of *F. oxysporum* and *V. dahliae* VCs to maintain leaf surface area and to prevent chlorophyll degradation seems to mitigate the adverse effect of salt stress on photosynthesis, thus helping sustain growth. Moreover, increased lateral root density in response to VC exposure would certainly help nutrient acquisition under unfavourable environmental conditions. Increased salt tolerance would benefit the growth of *F. oxysporum* and *V. dahliae* by ensuring the supply of root-derived nutrients such as root exudates and dead root cells and soil nutrient mobilisation by plants.

### Auxin signalling is involved in increased salt tolerance caused by V. dahliae VCs

**Figure 2.**
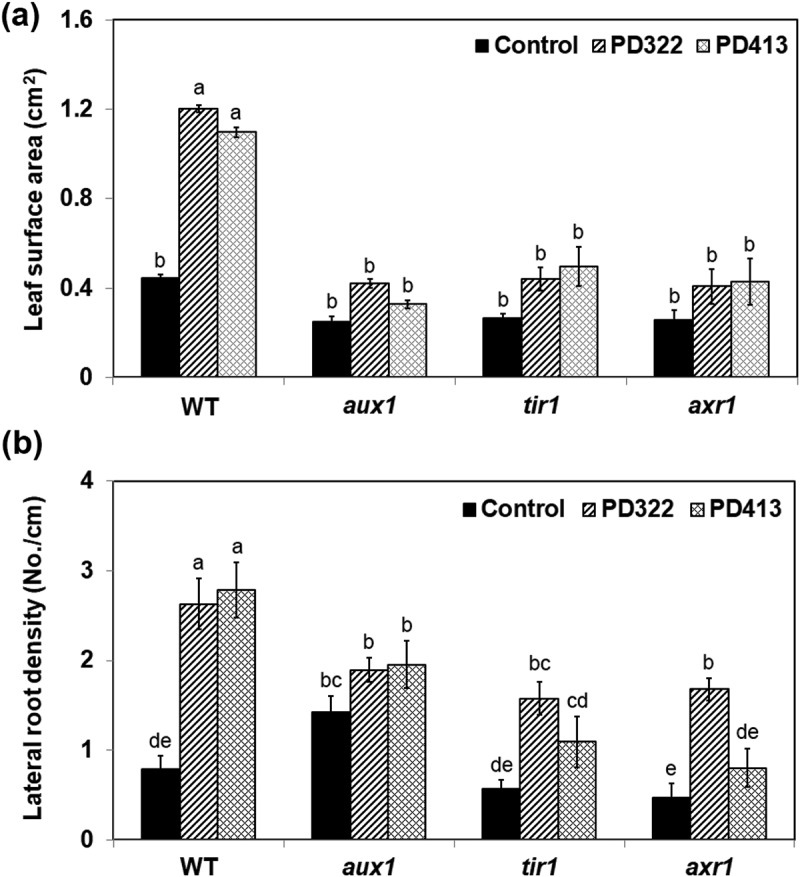
Growth responses of *A. thaliana* auxin signalling mutants to *V. dahliae* VCs in the presence of NaCl. Col-0 wild type (WT) and mutants of Col-0 defective in polar auxin transport (*aux1-*7) or auxin response (*tir1-1* and *axr1-3*) on medium containing 100 mM NaCl were co-cultivated with PDA only (control) and *V. dahliae* strains PD322 and PD413 for 14 days. Leaf surface area (a) and lateral root density (b) after treatment are shown. Values shown correspond to the mean ± SE of data from three replicates (*n* = 15). Different letters indicate significant difference among treatments, according to Fisher’s LSD test at *P* = 0.05.

Maintenance of plant hormone homeostasis protects plants from salt-induced damages (Hasanuzzaman et al. 2013; Deinlein et al. 2014). The involvement of auxin in plant stress tolerance has been well established (Fu and Wang 2011; Pieterse et al. 2012; Naseem et al. 2015; Forni et al. 2017). Based on previous studies showing the critical role of auxin signalling in controlling plant responses to VCs produced by various microbes (Zhang et al. 2007; Splivallo et al. 2009; Bailly et al. 2014; Bitas et al. 2015; Garnica-Vergara et al. 2016), we investigated how VCs produced by two *V. dahliae* strains affect the growth of *A. thaliana* mutants defective in auxin influx (*aux1-7*) or response (*tir1-1* and *axr1-3*) in the presence of 100 mM NaCl. These mutants were chosen because their growth responses to VCs produced by *F. oxysporum* and *V. dahliae* were defective (Bitas et al. 2015; Li et al. under revision). Upon exposure to *V. dahliae* VCs, the leaf surface area and lateral root density of all mutants did not increase as much as that of the wild type (Figure 2). In particular, both the leaf surface area and lateral root density of *aux1-7* did not look significantly different from those observed in control treatment. However, the *tir1-1* and *axr1-3* mutants displayed significantly increased lateral root densities when exposed to PD322 VCs compared to PD413 VCs and control treatment (Figure 2(b)). These results indicated the involvement of *AUX1, TIR1* and *AXR1* in mediating increased salt tolerance conferred by *V. dahliae* VCs, with *AUX1* playing a key role. It was reported that under high salt, the inhibition of lateral root formation is caused by suppression of basipetal auxin transport, resulting in over-accumulation of auxin in root epidermis of elongation zone while depleting auxin in root apex (Wang et al. 2009). *AUX1* encodes a non-redundant auxin influx carrier (Marchant 1999), which facilitates auxin distribution in lateral root cap and elongation of epidermal cells (Marchant et al. 2002; Band et al. 2014). The *aux1-7* mutant exhibited higher susceptibility to salt stress than the wild type, suggesting that auxin transport is required for alleviating salt stress (Wang et al. 2009; Galvan-Ampudia and Testerink 2011).

**Figure 3.**
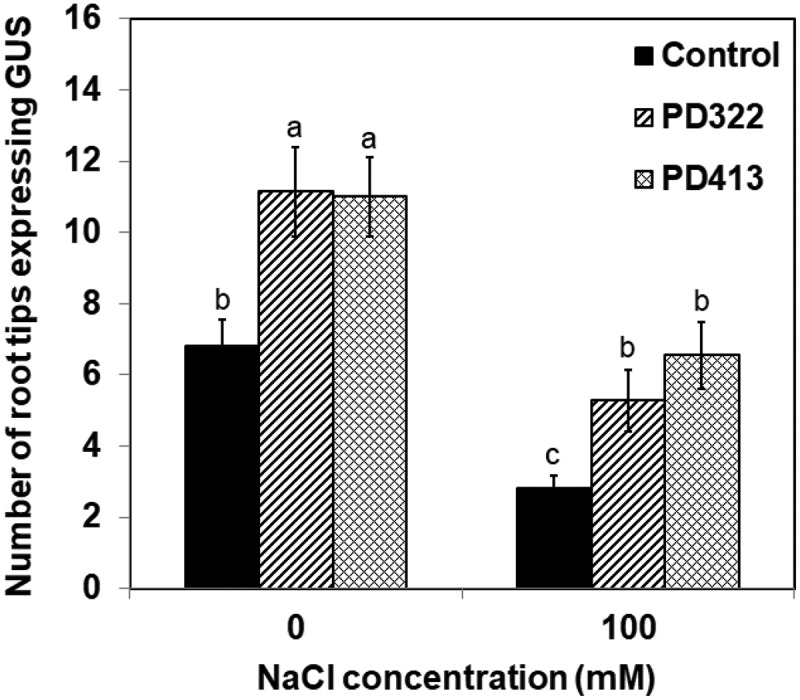
Expression of GUS in root tips of transgenic *A. thaliana* Col-0 containing *DR5::GUS*. Plants were stained for GUS activity after 7 days of co-cultivation with PDA only (control) and *V. dahliae* strains PD322 and PD413. Plants were cultured on medium containing no NaCl or 100 mM NaCl. The number of root tips expressing GUS for each treatment is shown. Values shown correspond to the mean ± SE of data from three replicates (*n* = 15). Different letters indicate significant difference among treatments, according to Fisher’s LSD test at *P* = 0.05.

We hypothesise that *V. dahliae* VCs increase salt tolerance by facilitating auxin recycling mediated by polar auxin transport. To test this hypothesis, we first determined whether increased auxin accumulation in root apex could be observed after VC treatment using a transgenic *A. thaliana* line that contains the auxin-responsive reporter *DR5::GUS*. This reporter consists of the *GUS* (β-glucuronidase) gene under the control of DR5, a highly active auxin-responsive synthetic promoter (Ulmasov et al. 1997). After 7 days of co-cultivation with *V. dahliae* in the presence of 100 mM NaCl, a greater number of root apex displayed pronounced GUS staining compared to control plants (Figure 3), indicating that increased auxin accumulation is associated with reduced inhibition of root growth in the presence of NaCl. A previous study showed that application of exogenous auxin led to increased length and cell number of root meristem in the presence of 100 mM NaCl (Liu et al. 2015).

**Figure 4.**
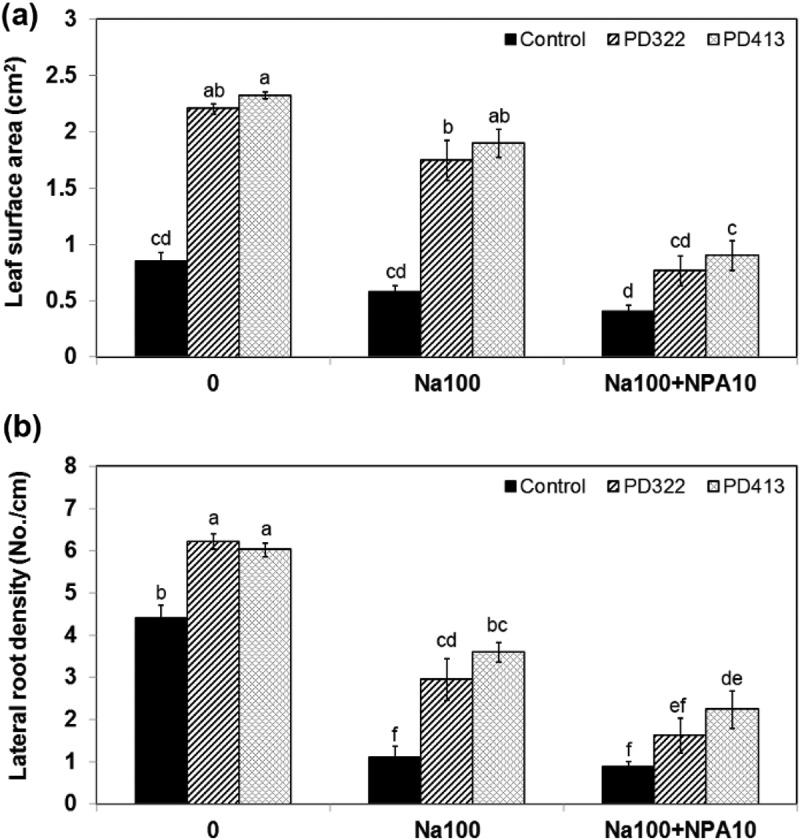
Growth response of *A. thaliana* to *V. dahliae* VCs in the presence of an inhibitor of polar auxin transport, 100 mM NaCl, or both. Leaf surface area (a) and lateral root density (b) of Col-0 seedlings co-cultivated with PDA only (control) and *V. dahliae* strains PD322 and PD413 for 14 days under three conditions, including no NaCl, 100 mM NaCl (Na100) and a combination of 100 mM NaCl and 10 μM NPA (Na100 + NPA10), are shown. The same volume of DMSO was used for all treatments. Values shown correspond to the mean ± SE of data from three replicates (*n* = 15). Different letters indicate significant difference among treatments, according to Fisher’s LSD test at *P* = 0.05.

To further investigate whether polar auxin transport plays an important role in robust root growth under salt stress caused by *V. dahliae* VCs, plants grown on medium containing NPA, which disrupts auxin recycling in *A. thaliana*, were exposed to *V. dahliae* VCs. The NPA treatment reduced the growth enhancement (Figure 4). The growth enhancement effect of PD322 VCs was completely blocked by NPA. However, PD413 VCs still increased plant growth in the presence of NPA. Analysis of VCs produced by PD322 and PD413 showed the production of both common and strain-specific compounds (Li et al. under revision). Six VCs, including 2-methyl-1-propanol, 3-octanone, 3-octanol, 1-octen-3-ol, himachala-2,4-diene and phenylethyl alcohol, were produced by both strains. PD322 emits two specific VCs (2-methyl-1-butanol and 4,11-selinadiene), while five VCs, including 3-methyl-1-butanol, 3-pentenol, 4-methyl-5-hexen-2-ol, 4-methyl-6-hepten-3-ol and 3,5,5-trimetyl-2-hexene, were uniquely produced by PD413. This finding suggested that the difference between the two *V. dahliae* strains may be caused by different VCs they produce.

### F. oxysporum VCs induced PR1 expression, but their effect on disease suppression varied depending on the strains used

**Figure 5.**
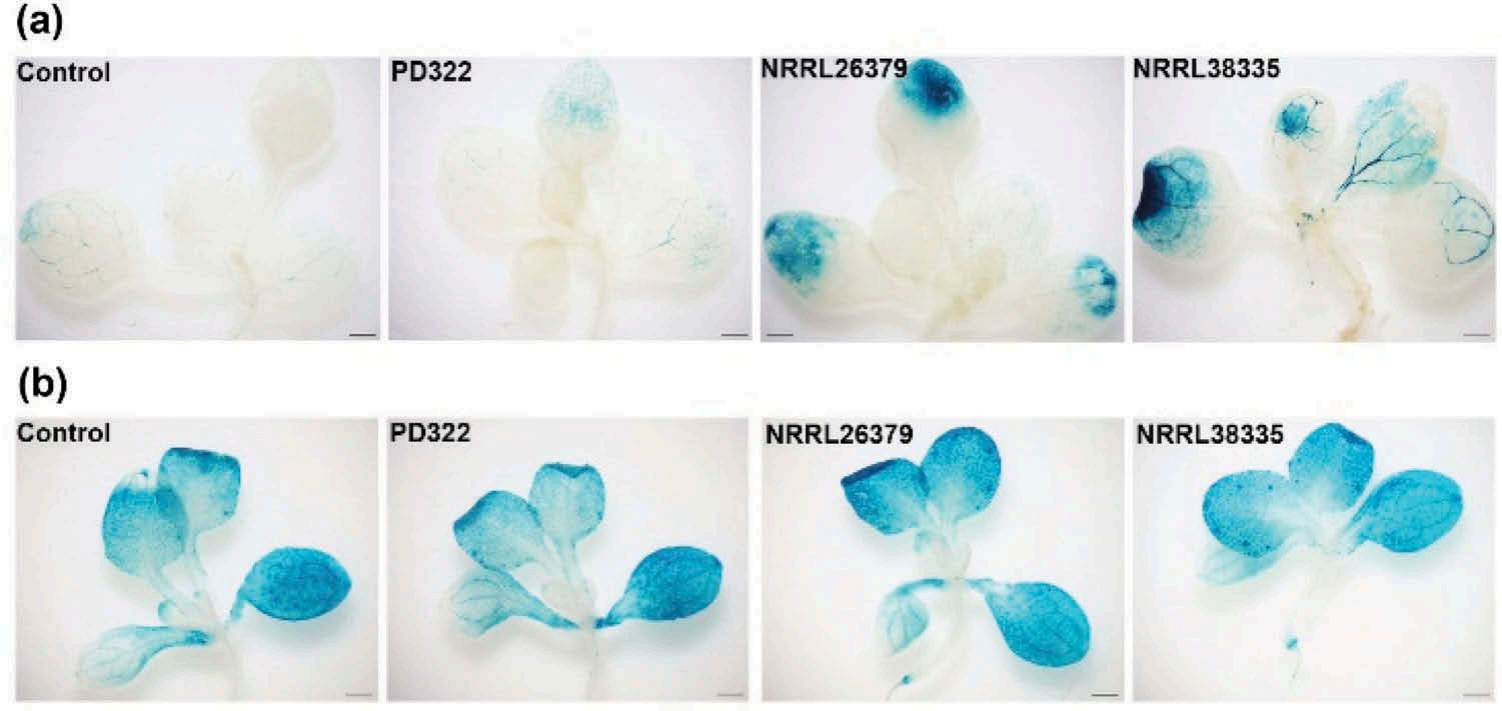
Expression of two defence-related genes in leaves of *A. thaliana* after exposure to fungal VCs. Transgenic *A. thaliana* Col-0 seedlings containing *PR1::GUS* (a) and *PDF1.2::GUS* (b), respectively, were co-cultivated with PDA only (control), *V. dahliae* (PD322) and *F. oxysporum* (NRRL26379 and NRRL38335) for 7 days. Blue staining indicates the activity of expressed GUS *in planta*. Scale bar = 500 μm.

Besides affecting growth and development, VCs produced by some microbes seem to function as elicitors for ISR (Bitas et al. 2013; Farag et al. 2013; Song and Ryu 2013; Chung et al. 2016; Li et al. 2016). To evaluate if VCs produced by *F. oxysporum* and *V. dahliae* also affect plant defence signalling, we employed two *A. thaliana* transgenic lines containing *PR1::GUS* and *PDF 1.2::GUS*, respectively. These reporters have been commonly used for monitoring the salicylic acid (SA)- and jasmonic acid/ethylene (JA/ET)-mediated defence signalling pathways, respectively. Compared to control plants and those exposed to *V. dahliae* PD322 VCs, *PR1::GUS* expression in plants exposed to VCs from NRRL26379 and NRRL38335 was noticeably higher after 7 days of co-cultivation (Figure 5(a)), suggesting the induction of the SA-dependent signalling pathway by *F. oxysporum* VCs. Similar to our finding, the induced expression of *PR1* was observed by VCs emitted by *Paenibacillus polymyxa* E681 and *T. asperellum* IsmT5 and by specific compounds (e.g. tridecane, *m*-cresol and methyl benzoate) (Lee et al. 2012; Naznin et al. 2014; Kottb et al. 2015). However, our analysis of VCs produced by these *F. oxysporum* strains did not reveal the production of these compounds (Bitas et al. 2015), suggesting the involvement of different compounds in activating the expression of *PR1*. Unlike the expression of *PR1::GUS, PDF1.2::GUS* was highly expressed in whole leaves of both control and fungal VC-treated plants (Figure 5(b)). Previous studies reported that VCs emitted by several plant growth promoting fungi, including *Ampelomyces* sp., *Cladosporium* sp. and *Trichoderma* sp., reduced disease symptoms through the activation of signalling pathways that involve SA and JA/ET (Naznin et al. 2014; Kottb et al. 2015). However, Ryu et al. (2004) reported that ISR elicited by VCs emitted by *B. amyloliquefaciens* GB03 was mediated by ET signalling but did not require the SA or JA signalling pathway. Different responses of *A. thaliana* to VCs from different microbes could depend on the type and amount of volatile elicitors released by individual microbes.

**Figure 6.**
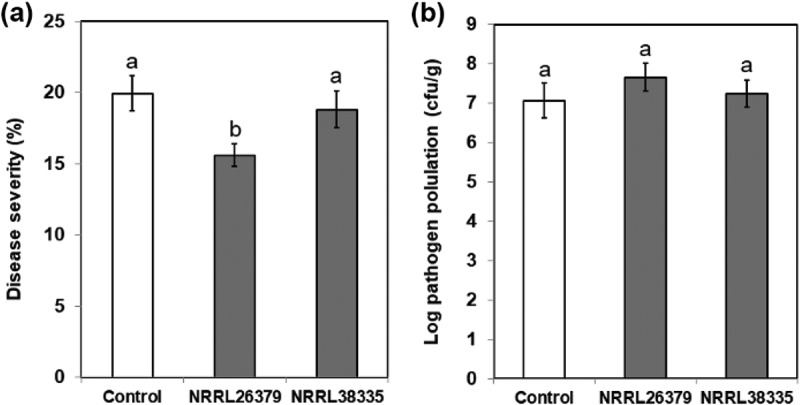
Effect of *F. oxysporum* VCs on *A. thaliana* defence against *P. syringae*. After co-cultivating Col-0 seedlings with PDA only (control) and *F. oxysporum* (NRRL26379 and NRRL38335) for 12 days, the seedlings were inoculated with *Pst* DC3000. Disease severity (a) was scored as the percentage of total leaf surface with symptoms, ranging from 0 (no symptoms) to 100 (complete chlorosis or necrosis). The number of bacterial cells in inoculated leaves (b) under each treatment is shown. Values shown correspond to the mean ± SE of data from three replicates (*n* = 15). Different letters indicate significant difference among treatments, according to Fisher’s LSD test at *P* = 0.05.

To investigate whether the activation of the SA signalling pathway by *F. oxysporum* VCs causes enhanced resistance to *P. syringae*, we infected *A. thaliana* with *Pst* DC3000. To eliminate the possibility that *F. oxysporum* VCs directly suppress *P. syringae*, we removed *F. oxysporum* culture from I plate prior to pathogen inoculation. VCs from NRRL26379 significantly reduced disease severity compared to control (Figure 6(a)). However, no such effect was observed upon exposure to NRRL38335 VCs. To determine if reduced disease symptom was due to reduced bacterial density in infected leaves, the number of bacterial cells in plant apoplast was quantified. The bacterial cell density after the exposure to *F. oxysporum* VCs did not look significantly different from that in control plants (Figure 6(b)). These results suggested that preexposure to VCs produced by NRRL26379 could reduce disease severity caused by *P. syringae* infection without suppressing the pathogen population in the leaves. Further studies are needed to understand the molecular mechanism underlying reduced disease severity caused by NRRL26379 VCs. A comprehensive analysis of gene expression profiles in response to VCs from various fungal species and strains and the evaluation of additional *A. thaliana* mutants defected in different hormonal pathways will help explore this mechanism.

## Conclusion

Plants and microbes have co-evolved and employ diverse strategies to affect each other. Some microbes have been shown to enhance the ability of plants to manage abiotic and biotic stresses by releasing phytohormones, siderophore and/or other small molecules (Ortíz-Castro et al. 2009; Vaishnav et al. 2013). In return, microbes may receive nutrients and better protected ecological niche. Recently, a number of studies suggested the role of microbial VCs in improving plant’s ability to manage different types of stress, including drought, salinity and pathogen infection (Ortíz-Castro et al. 2009; Bailly and Weisskopf 2012; Bitas et al. 2013; Farag et al. 2013; Kanchiswamy et al. 2015; Chung et al. 2016; Li et al. 2016; Piechulla et al. 2017). Here, we showed that VCs produced by soilborne fungal pathogens *F. oxysporum* and *V. dahliae* also help *A. thaliana* growth better in the presence of salt. The growth response of *A. thaliana* auxin signalling mutants, auxin response monitored using a reporter gene, and the effect of inhibition of auxin transport suggested that some of its components are needed to confer fungal VC-mediated enhanced salt tolerance. Further studies are needed to evaluate whether fungal VCs affect stress tolerance of plants under field conditions. Further elucidation of the molecular mechanism underlying VC-mediated effects on plants may present a novel strategy for better managing crop growth under biotic and abiotic stresses.
